# Influence of calcination temperature and particle size distribution on the physical properties of SrFe_12_O_19_ and BaFe_12_O_19_ hexaferrite powders

**DOI:** 10.1038/s41598-024-67994-8

**Published:** 2024-07-30

**Authors:** Jakub Hlosta, Kamila Hrabovská, Jiří Rozbroj, Jan Nečas, David Žurovec, Jan Diviš, Ondřej Životský

**Affiliations:** 1 Faculty of Mining and Geology, VSB - Technical University of Ostrava, 17. listopadu 2172/15, 708 00 Ostrava, Czech Republic; 2 Faculty of Electrical Engineering and Computer Science, VSB - Technical University of Ostrava, 17. listopadu 2172/15, 708 00 Ostrava, Czech Republic

**Keywords:** M-type ferrites, BaFe_12_O_19_, SrFe_12_O_19_, Microstructure, Magnetic interactions, Hysteresis loops, Chemical engineering, Ferromagnetism, Magnetic properties and materials

## Abstract

The paper deals with the economic optimisation of ferrite powder preparation during producing hard ferrite magnets. The magnetic properties of ferrites are investigated by replacing feedstock and reducing calcination temperature and particles in the order of tens of microns. The granulates about 8–10 mm in size were calcined for 2 h in the temperature range from 1100 °C to 1300 °C and additionally crushed and milled to an average particle size of about 80–90 µm. The scanning electron microscopy images confirmed the agglomerates of particles with different shapes and sizes in tens of µm. The X-ray diffraction measurements revealed that, besides the SrFe_12_O_19_ and BaFe_12_O_19_ phases, there was also the presence of 2–39% hematite. The highest values of maximum energy product (BH)_max_ = 930 J/m^3^ and remanent magnetic induction B_r_ = 72.8 mT were obtained at a calcination temperature of 1300 °C. The Henkel plots confirmed the presence of exchange-coupling and dipolar magnetic interactions at lower and higher magnetic fields, respectively. The strength of interactions was also dependent on the calcination temperature. Replacing strontium with barium led to a deterioration of the magnetic parameters, which were optimal at a lower calcination temperature (1100 °C). This phenomenon was partly overcome by reducing the mean particle size of Ba-based hexaferrites to 45–50 µm.

## Introduction

Magnets have many uses in various fields, and their worldwide consumption is increasing. Permanent magnets find applications in the automotive, electrical and energy industries, medical devices, and others^1–9^. The most used permanent magnets are hard ferrite magnets^10^. It is because they are the most affordable. In addition to the wide-spread barium ferrites, highly coercive strontium ferrites are increasingly used. Barium and strontium hexaferrite magnets belong to permanent magnets with hexagonal structures^11^, which have desirable ferromagnetic properties. These hard hexaferrite magnets with the chemical formula BaFe_12_O_19_ and SrFe_12_O_19_, respectively, have good chemical stability, high Curie temperature and good corrosion resistance, and therefore, surface coating is not necessary before application^12^. Another advantage is the relatively lower cost than other permanent magnets, such as rare earth magnets, AlNiCo or SmCo. Hard hexaferrite magnets are currently being investigated as potential magnetic materials without rare-earth elements (RE) in medium energy product applications due to the need for new and cheaper magnetic materials with reduced or even no RE because of the significant criticism of RE^13^.

Because of the unique combination of BaFe_12_O_19_ magnetic properties, chemical and thermal stability along with suitable applications in modern technologies, several of their physical properties, such as magnetoelectric^14^, multiferroic^15^ and microwave absorption^16^, have been studied. The low cost of BaM, good availability and relatively easy processing are also advantages. Nanoscale hexagonal ferrites are also promising materials for the fabrication of a new generation of permanent magnets, as well as high-density data recording and storage systems^17^. The mechanical and physical properties of powdered materials have an overall impact on their handling and processing behaviour^18,19^.

Generally, the properties of ferrites depend strongly on their composition, synthesis conditions, crystallinity, shape, particle size and distribution^20–23^. The calcination temperature of the reaction usually ranges between 1250 and 1400 °C^24^. Another study dealing with calcination temperature’s effect found that 1100 °C is a suitable temperature^24^. Their SEM photographs showed grains in the shape of regular hexagonal plates. In addition, maximum saturation magnetization was obtained at 1200 °C. However, the coercivity of the synthesized BaFe_12_O_19_ samples was lower than the theoretical values^24^. Another study shows a broader temperature range from 800 °C to 1100 °C. The effect on particle morphology, particle size, magnetic hysteresis and optical properties was studied^25^. The impact of not only temperature but also the calcination time of the powders was found. Extending the calcination time to 24 h led to the forming a fully crystalline BaM phase at 1100 °C. Extending the calcination time to 124 h led to thermal decomposition and formation of BaO as the second phase. The magnetic domain structure transformed from single to multi-domain with increasing temperature from 1100 °C to 1300 °C^10^.

The formation of hexagonal ferrites is a highly complicated process, and the underlying mechanisms are still not fully understood. However, they have been of interest for many researchers worldwide for over 50 years^26–30^. Therefore, in this study, the effect of calcination temperature and particle size distribution of ground calcite on the magnetic properties of M-type ferrite powders was investigated. The quality of input raw materials mainly influences the calcination process, the molar ratio (SrO:Fe_2_O_3_) ~ 5.85, the size of the granulate, the temperature profile of the calcination furnace, the furnace flow rate and the processes taking place in the cooling retort of the furnace.

The whole research was carried out in cooperation and according to the requirements of the manufacturer of hard ferrite magnets. The study shows the development and optimization of the technology of mixing basic raw materials and grinding of ferrite calcite, determination of the influence of granulometric and frictional parameters of feedstock and setting of preparation technology of ferrite dust as a feedstock for the production of hard ferrite magnets. The aim was to ensure constant and quantifiable outputs from the different parts of the process line, which would improve the final product’s magnetic properties. The findings obtained in a partial study of the effect of calcination temperature and particle size distribution on the magnetic properties of SrFe_12_O_19_ and BaFe_12_O_19_ ferrite powders are presented.

Many publications have focused on improving ferrites’ magnetic, chemical and mechanical properties by increasing calcination temperatures or using finer particles down to the nanometer range. This entails a significant increase in the technological and economic requirements of the process. The main contribution of this study should be to provide an overview of an economically suitable setup usable for routine and sustainable mass production as well as small-scale production. There are a multitude of synthesis methods and technological setups. Thus, each study is unique and has its significance. Therefore, even today, publications are still being published on this topic.

## Materials and methods

### Input powders characterization

Iron oxide (Fe_2_O_3_), strontium carbonate (SrCO_3_), and barium carbonate (BaCO_3_) were used as input powders for the preparation of hexagonal ferrites. The basic mechanical and physical properties of input powders are shown in Table 1, and the chemical compositions available from the manufacturers are presented in Table 2. Freeman FT4 Powder Rheometer was used to determine the flow properties and compressibility. The flow properties are significant due to the homogenization of both compounds and the compression process used to produce hard ferrite magnets. The particle size distribution was determined by laser diffraction using a CILAS 1190 Particle Size Analyzer (wet method).

**Table 1:** Mechanical and physical parameters of the compounds used.

Parameter	Symbol	Unit	Fe_2_O_3_	SrCO_3_	BaCO_3_	Sr_MIX	Ba_MIX
Angle of internal friction	*φ*	(°)	35.6	39.2	40.4	34.7	35.1
Angle of internal friction (ef.)	*φ*_e_	(°)	47.8	56.7	49.9	42.9	44.3
Cohesion	*τ*_0_	(kPa)	3.08	4.93	3.11	2.15	2.83
Unconfined yield strength	*σ*_c_	(kPa)	12.0	20.8	13.4	8.21	8.98
Major principal stress	*σ*_1_	(kPa)	27.4	34.5	35.6	26.7	27.7
Flow function	*ff*_c_	(–)	2.29	1.66	2.65	3.25	2.01
Bulk density	*ρ*_b_	(g/cm^3^)	0.53	0.71	1.38	0.60	0.86
Compressibility (15 kPa)	*C*_15kPa_	(%)	25.4	37.2	23.1	18.4	19.7
Particle size (10%)	*d*_10_	(μm)	0.79	1.20	1.04	0.60	0.54
Particle size (50%)	*d*_50_	(μm)	13.81	4.34	4.19	12.36	11.89
Particle size (90%)	*d*_90_	(μm)	35.78	11.82	11.26	35.57	33.12
Particle size (avg.)	*d*	(μm)	16.47	5.56	5.35	15.81	13.56

**Table 2:** Chemical composition of compounds used (wt%).

Iron oxide		Strontium/barium carbonate	
Fe_2_O_3_	Min. 99.0%	SrCO_3_/BaCO_3_	Min. 98.0%
MnO	Max. 0.30%	BaO	Max. 1.90%
SiO_2_	Max. 0.05%	CaO	Max. 0.17 %
Al_2_O_3_	Max. 0.10%	Na_2_O (+ K_2_O)	Max. 0.10%
Cr_2_O_3_	Max. 0.03%	Fe_2_O_3_	Max. 0.006%
CaO	Max. 0.04%	Al_2_O_3_	Max. 0.01%
MgO	Max. 0.02%	SO_3_	Max. 0.40%
CuO	Max. 0.02%		
SO_3−_	Max. 0.10%		
Cl_−_	Max. 0.15%		
Na_2_O + K_2_O	Max. 0.04%		

The iron oxide is a deep red powder with a mean grain size of 16.5 μm and spherical particle shape with sharp-edged chipped fragments. The flow function is defined as the ratio of the principal normal stress to the interstitial strength of the powder. It determines the cohesiveness or, conversely, the ability to flow freely. Iron oxide can be classified as a cohesive powder according to its flow function. Since cohesive properties are often associated with average compressibility, Fe_2_O_3_ showed moderate compressibility during testing, typical of most powders. Strontium carbonate and barium carbonate are white powders with a rod-shaped grain size of approximately 5.5 μm in the middle. These small particles make the SrCO_3_ and BaCO_3_ samples very cohesive (ff_c_ < 2) and form agglomerates. SrCO_3_ exhibits the highest internal friction angles, cohesiveness and ultimate strength and the worst flow characteristics of all tested samples. The very fine particles also cause a high compressibility of 37.2% at a normal stress of 15 kPa.

Finally, two mixture samples denoted Sr_MIX and Ba_MIX were prepared in a given weight ratio of Fe_2_O_3_:SrCO_3_ (86.35:13.65) and Fe_2_O_3_:BaCO_3_ (88.74:17.52), respectively. A molar ratio of Fe_2_O_3_/SrCO_3_ (BaCO_3_) of 5.85 ± 0.1 was used. The weight ratio of input materials is based on the technological specification of the collaborating manufacturer of hard ferrite magnets. These two feedstock mixtures were subsequently used to produce two types of strontium- and barium-based ferrite powders. Mixing input powders improves the flow properties, with the smaller SrCO_3_ and BaCO_3_ particles enveloping the larger Fe_2_O_3_ particles and acting as an additive to promote flow. This reduces the internal friction angle and compressibility while filling the interparticle space more efficiently. Characterization of the mixtures is essential from a processing point of view. Shear and flow properties could be important in homogenization and handling processes during production. The mechanical and physical properties of the mixtures are compared to the input powders in the last two columns of Table 1.

**Figure 1:**
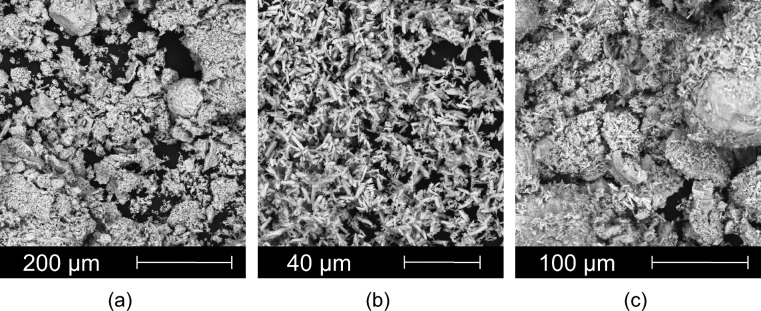
SEM micrographs of input powders: (**a**) Fe_2_O_3_; (**b**) SrCO_3_; (**c**) Sr_MIX (Fe_2_O_3_:SrCO_3_).

The SEM images (FEI Quanta 650 FEG scanning electron microscope) of Fe_2_O_3_, SrCO_3_ and their mixtures are presented in Fig. 1. The micrographs show larger Fe_2_O_3_ particles with a wider particle size distribution and needle-like smaller SrCO_3_ particles. The mixture image confirms a uniform and homogeneous distribution of both components, with smaller particles adhering to the surface of the larger particles. The formed agglomerates are important for the chemical reactions during calcination. A similar microstructure is detected for the Fe_2_O_3_:BaCO_3_ mixture.

### Samples preparation

**Figure 2:**
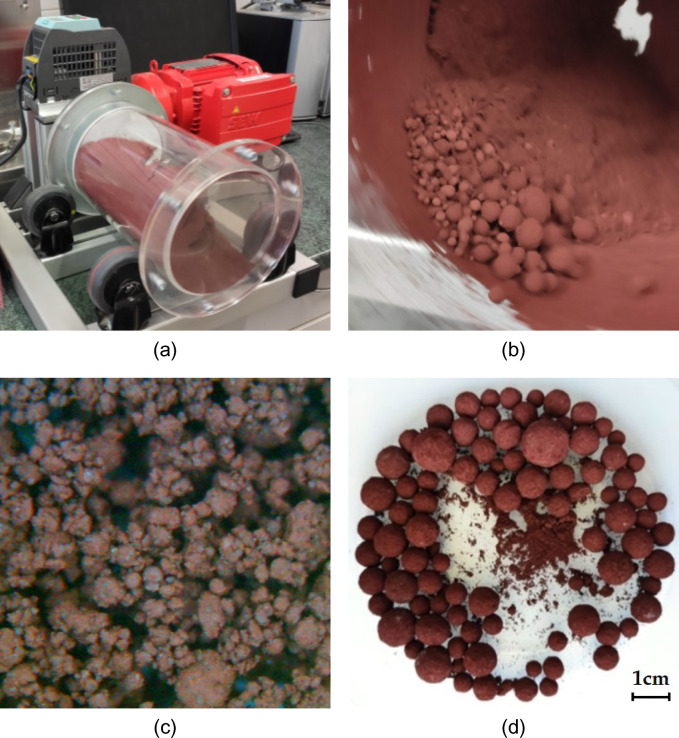
Ferrite powder samples preparation: (**a**) homogenization; (**b**) granulation; (**c**) granulate structure; (**d**) granules.

Figure 2 shows the preparation process of ferrite powders. A laboratory rotary drum with a diameter of 140 mm and a length of 250 mm was used for mixing and granulation, with a speed of approximately 60 rpm. During granulation, water was gradually added in the form of aerosol. The resulting granulates with a 20–23% moisture content were dried freely in the air for one day. The granulates were calcined in a laboratory oven and preheated to the desired sample temperature (1100–1300 °C)—the remaining moisture content evaporated during the calcination process. The calcination time was set to 2 h, after which the samples were cooled freely in the oven to ambient temperature. After calcination, the samples were crushed and milled with a laboratory grinder to the required granulometry, according to Table 3.

**Table 3:** Prepared strontium- and barium-based ferrite powders; calcination temperature *T*_c_, particle size distributions *d*_10_, *d*_50_, *d*_90_, *d*_*avg*_ and density *ρ*.

Sample	*T*_c_ (°C)	*d*_10_ (µm)	*d*_50_ (µm)	*d*_90_ (µm)	*d*_*avg*_ (µm)	*ρ* (g/cm^3^)
Sr_4.1	1150	56.52	82.46	120.26	85.38	5.27
Sr_5.1	1200	50.23	79.42	118.99	81.79	5.14
Sr_1 (ref)	≈ 1250	28.80	67.31	157.29	82.73	5.25
Sr_6.1	1250	50.95	83.03	142.42	91.83	5.11
Sr_7.1	1300	31.00	74.42	151.07	84.92	5.12
Ba_1.2	1100	15.15	52.81	126.05	63.17	5.39
Ba_3.2	1200	16.49	78.79	144.34	75.79	5.33
Ba_4.2	1250	24.73	86.18	152.66	87.86	5.30
Ba_5.2	1300	29.50	93.24	155.49	93.63	5.33
Ba_1	1100	21.52	43.03	68.57	45.18	5.36
Ba_2.1	1150	24.07	43.41	67.52	45.19	5.33
Ba_3.1	1200	18.48	50.32	83.34	51.03	5.30
Ba_4.1	1250	20.14	43.03	70.56	44.53	5.30
Ba_5.1	1300	16.93	49.54	74.37	48.63	5.31

### Characterization techniques

The X-ray diffraction (XRD) measurements were performed on a Bruker-AXS D8 Advance (Germany) in 2Θ/Θ geometry with a position-sensitive LynxEye detector under the following conditions: CuKα/Ni filter radiation, 40 kV voltage, 40 mA current, step mode with 0.014° 2Θ step with a total time per step of 2 s (summation of five measurements with 0.25 s step) and digital processing of the resulting data. The Bruker Diffrac Suite software was used for measurements and data evaluation. The quantification was based on the Rietveld method of structural analysis from powder diffraction data. It consisted of modelling the diffraction spectra using known structural data (lattice parameters, positions of atoms in the structure, spatial group, occupancy factors, etc.). Input structural data were taken from the Bruker DiffracPlus Topas structure database (hematite), from the COD database (Crystallographic Open Database—SrFe_12_O_19_), and the American Mineralogist crystal structure database (BaFe_12_O_19_).

An FEI Quanta-650 FEG auto-emission electron microscope from FEI (Thermo Fisher Scientific) was used for photographic documentation and identifying individual minerals. The imaging was performed using a back-scattered electron detector (BSED) in chemical gradient mode at 10 kV voltage and 4.5–5 µm beam diameter. Anton Paar Ultrapyc 5000 gas pycnometer was used for powder density determination.

Magnetic measurements using a MicroSense EZ9 vibrating sample magnetometer (VSM) were performed to confirm and characterise the ferromagnetic state in the studied samples. The measured magnetization curves at room temperature show the dependence of the magnetic polarization *J* of the sample on the external magnetic field *H* (*J–H* curves). The maximal applied magnetic field was ± 1600 kA/m (± 2 T). We also plotted *B–H* curves representing the dependence of the magnetic induction *B* on the external magnetic field *H*. The following magnetic parameters were obtained from the J–H and B–H curves: the maximum energy product, (*BH*)_max_; remanent magnetic induction, *B*_r_; coercivity of the *J-H* curve, *H*_cJ_; coercivity of the *B-H* curve, *H*_cB_. The interparticle magnetic interactions of prepared powders were analysed using the Henkel plot (Δ*M* function). It describes the relationship between the virgin *M*_vir_ (*H*) and the magnetization curve *M*(*H*) using the relation:

1 $$\Delta M\left( H \right) = M_{vir} \left( H \right) - 0.5 \cdot \left( {M_{up} \left( H \right) + M_{down} \left( H \right)} \right)$$

where *M*_up_(H) and *M*_down_(H) are magnetizations in increasing and decreasing positive magnetic field *H*. Demagnetization of the samples takes place in an alternating magnetic field with an exponential amplitude decrease coefficient of 0.95.

## Results and discussion

**Figure 3:**
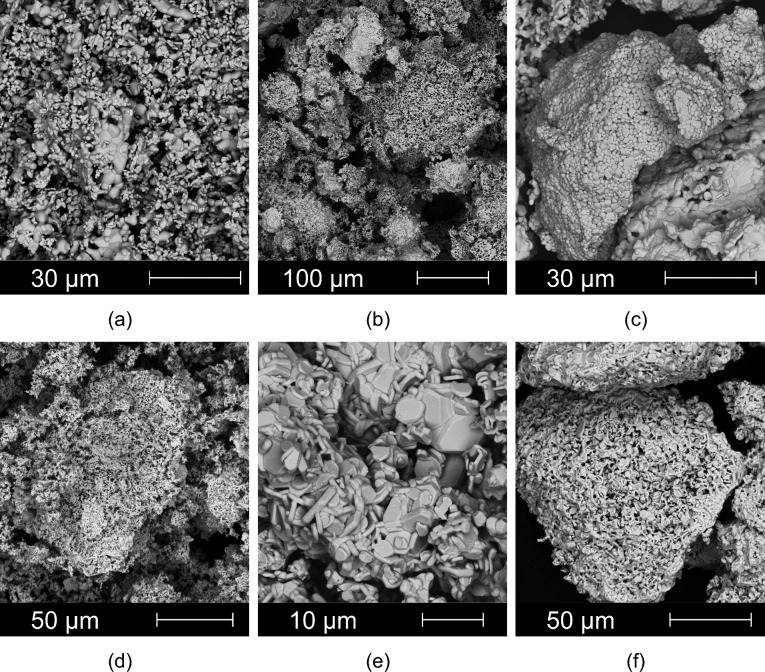
SEM micrographs of selected SrFe_12_O_19_ and BaFe_12_O_19_ hexaferrites: (**a**) Sr_1; (**b**) Sr_4.1; (**c**) Sr_7.1; (**d**) Ba_1.2; (**e**) Ba_5.1; (**f**) Ba_5.2.

Table 3 lists all the samples of SrFe_12_O_19_ and BaFe_12_O_19_ ferrite powders that were prepared for physical properties testing. The reference sample Sr_1 was taken directly from the research company production and characterised the current SrFe_12_O_19_ production technology. Figure 3a–f shows the SEM morphology of chosen SrFe_12_O_19_ and BaFe_12_O_19_ samples calcined for 2 h at different calcination temperatures. The presence of large and small hexagonal-like platelet particles, in addition to semi-circular polyhedral particles, was observed. The agglomerates contained a wide distribution of particles with sizes between 1 and 150 μm), and the average particle size was about 70 to 90 (m) (top and middle part of Table 3). Due to the deterioration of the magnetic properties, Ba-based hexaferrites were also prepared with a lower average particle size of around 45–50 μm (lower part of Table 3).

**Figure 4:**
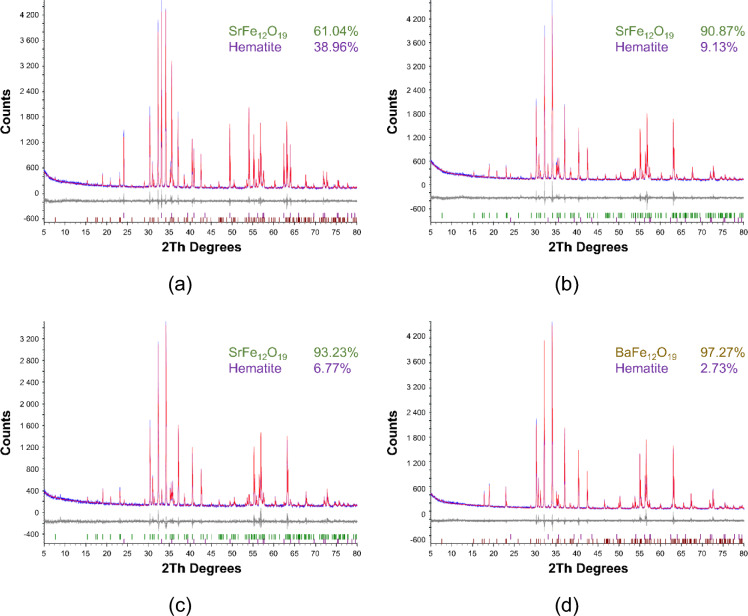
XRD diffractograms of selected hexaferrite samples: (**a**) Sr_1; (**b**) Sr_6.1; (**c**) Sr_7.1; (**d**) Ba_5.1.

The XRD patterns of selected SrFe_12_O_19_ and BaFe_12_O_19_ ferrite powders are shown in Fig. 4, and the results of the Rietveld analysis of all samples are summarized in Table 4. The diffractograms confirm the existence of two main phases: SrFe_12_O_19_ (BaFe_12_O_19_) and Fe_2_O_3_. All prepared Ba-based hexaferrites contain only about 2 wt% of hematite, which confirms their high structural homogeneity. In the case of Sr-based samples, unreacted hematite is more abundant. The reference sample exhibited a high percentage of hematite with a content of about 39 wt%. On the contrary, the laboratory-prepared Sr samples showed a lower hematite concentration ranging between 7 and 12%, weakly dependent on the calcination temperature. The reason is a different way of calcination of the reference sample that was placed in a rotary oven heated to 1430 ± 20 °C and calcined for 45 ± 5 min, during which the temperature gradually decreased to 1000 °C. Finally, the sample was cooled down to room temperature inside the oven. To optimise and reduce the energy consumption of the industrial plant, the research objective was to investigate the behaviour of the hematite content for temperatures up to 1300 °C. Therefore, the calcination temperature of the reference sample cannot be precisely determined and should range around the mean value of 1250 °C. These results clearly show the high sensitivity of Sr hexaferrite structure to the calcination procedure.

**Table 4:** Structural data of the strontium- and barium-based ferrite powders; calcination temperature *T*_c_, mean particle size *d*_*avg*_ and lattice parameters *a* and *c* obtained from the Rietveld analysis.

Sample	*T*_c_ (°C)	*d*_*avg*_ (µm)	Hematite			SrFe_12_O_19_/BaFe_12_O_19_		
			Content (wt%)	*a* (Å)	*c* (Å)	Content (wt%)	*a* (Å)	*c* (Å)
Sr_4.1	1150	85.38	11.81	5.270	13.747	88.19	5.882	23.050
Sr_5.1	1200	81.79	6.97	5.036	13.747	93.03	5.883	23.051
Sr_1 (ref)	≈ 1250	82.73	38.96	5.035	13.748	61.04	5.882	23.050
Sr_6.1	1250	91.83	9.13	5.036	13.746	90.87	5.882	23.049
Sr_7.1	1300	84.92	6.77	5.036	13.745	93.23	5.882	23.052
Ba_1.2	1100	63.17	2.06	5.033	13.752	97.94	5.893	23.201
Ba_3.2	1200	75.79	2.61	5.035	13.754	97.39	5.893	23.201
Ba_4.2	1250	87.86	1.41	5.029	13.781	98.59	5.893	23.203
Ba_5.2	1300	93.63	2.43	5.022	13.757	97.57	5.893	23.202
Ba_1	1100	45.18	2.52	5.033	13.761	97.48	5.893	23.201
Ba_2.1	1150	45.19	3.18	5.034	13.776	96.82	5.893	23.201
Ba_3.1	1200	51.03	3.15	5.032	13.757	96.85	5.893	23.202
Ba_4.1	1250	44.53	1.36	5.032	13.767	100.00	5.893	23.202
Ba_5.1	1300	48.63	2.73	5.034	13.758	97.27	5.893	23.203

**Figure 5:**
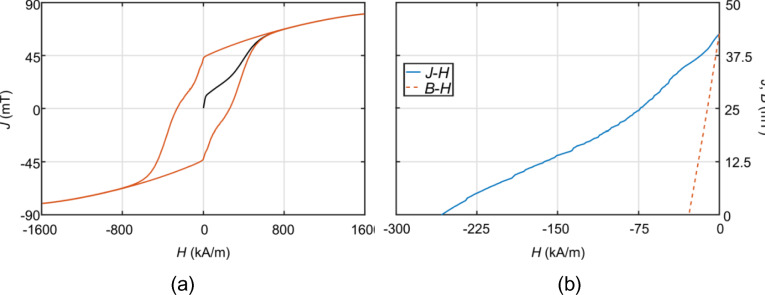
*J–H* and *B–H* curves of the Sr_4.1 sample: (**a**) complete *J–H* curve measured at high magnetic fields (± 1600 kA/m) including the virgin curve; (**b**) *J–H* and *B–H* demagnetization curves (the second quadrant).

**Figure 6:**
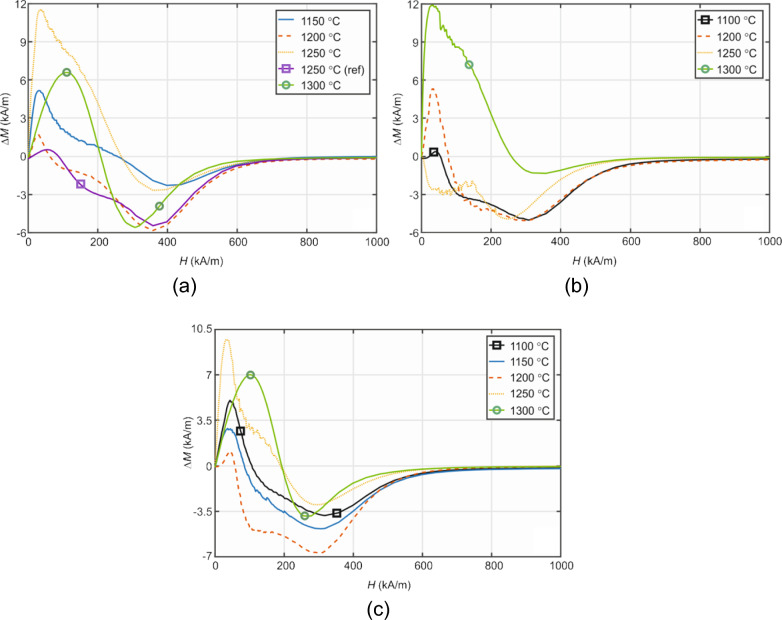
Henkel plots of prepared samples in dependence on the calcination temperature: (**a**) Strontium ferrite powders with the average particle size *d* = 80–90 µm; (**b**–**c**) Barium ferrite powders with *d* = 80–90 µm and 45–50 µm, respectively.

The magnetic properties of prepared samples are presented in Figs. 5 and 6. Figure 5a and b show an example of *J-H* and *B-H* curves measured for strontium ferrite sample (Sr_4.1) calcined at 1150 °C with the average particle size of 80–90 μm, respectively. Henkel plots of strontium ferrite are shown in Fig. 6a. Henkel plots of barium ferrite are shown in Fig. 6b and c. The shape of both magnetization curves is similar for all powder samples, and the magnetic parameters obtained are summarized in Table 5. In the case of Sr-based samples, it can be seen that the parameters determined from the *B–H* curve − (*BH*)_max_, *B*_r_, *H*_cB_—increase with increasing calcination temperature. The *H*_cj_ has the opposite tendency at and above 1250 °C. Therefore, high (*BH*)_max_, *B*_r_ and *H*_cB_ values are observed for Sr_7.1 sample calcined at 1300 °C, however, the *H*_cj_ value (~ 208 kA/m) is a little lower compared to the other samples. The reference sample Sr_1 calcined at 1250 °C shows the highest *H*_cj_ value, but the lowest saturation magnetization *M*_S_ due to the high amount of hematite detected by XRD measurements. If the calcination temperature exceeds 1300 °C, the magnetic properties of all parameters markedly deteriorate. Generally, the values of the magnetic parameters of laboratory-prepared micron-sized SrFe_12_O_19_ powders are comparable to those presented in the literature, e.g.^31^. However, better hard magnetic properties can be obtained if nanoparticles are used ^31^. Other published studies have confirmed the improvement of magnetic and crystallization properties with increasing calcination temperature^32,33^. This is also during low temperature synthesis^34,35^.

**Table 5:** Measured magnetic parameters of strontium- and barium-based ferrite powders in dependence on calcination temperature, e.g. (*BH*)_max_—maximum energy product, *B*_r_—remanent magnetic induction, *H*_cB_—coercivity of the *B–H* curve, *H*_cJ_—coercivity of the *J–H* curve, *M*_S_—saturation magnetization, Δ*M*_max_, Δ*M*_min_—the strongest positive and negative magnetic interactions.

Sample	Calcination temperature (°C)	(*BH*)_max_ (J/m^3^)	*B*_r_ (mT)	*H*_cB_ (kA/m)	*H*_cJ_ (kA/m)	*M*_S_ (Am^2^/kg)	Δ*M*_max_ (kA/m)	*H*_max_ (kA/m)	Δ*M*_min_ (kA/m)	*H*_min_ (kA/m)
Sr_4.1	1150	289	42.6	28.4	256.1	58.19	5.2	31.9	− 2.3	407.8
Sr_5.1	1200	632	60.2	39.6	265.5	64.54	1.7	30.3	− 5.8	358.1
Sr_1 (ref.)	1250	665	59.3	44.3	289.5	39.27	0.5	54.1	− 5.5	358.1
Sr_6.1	1250	567	59.1	39.6	186.1	63.05	11.5	38.2	− 2.7	358.1
Sr_7.1	1300	930	72.8	50.8	208.2	62.86	6.6	109.8	− 5.6	307.2
Ba_1.2	1100	502	51.7	38.0	217.9	65.69	0.4	44.6	− 5.0	308.8
Ba_3.2	1200	507	52.5	36.5	138.3	65.49	5.3	33.4	− 5.1	296.1
Ba_4.2	1250	428	58.2	28.4	109.6	65.61	− 0.1	0.0	− 4.9	245.1
Ba_5.2	1300	303	47.0	26.8	119.2	65.84	11.9	33.4	− 1.3	358.2
Ba_1	1100	465	50.1	36.5	195.6	65.38	5.0	43.0	− 3.8	318.3
Ba_2.1	1150	538	53.7	39.6	192.3	65.01	2.8	44.6	− 4.8	305.6
Ba_3.1	1200	638	58.5	42.7	192.3	65.40	1.1	44.6	− 6.7	300.8
Ba_4.1	1250	628	65.2	38.0	108.0	65.36	9.7	33.4	− 3.0	297.6
Ba_5.1	1300	722	63.4	44.3	176.4	65.27	7.0	101.9	− 3.9	265.8

The barium-based ferrites with an average particle size comparable to strontium samples are shown in the middle part of Table 5. Surprisingly, optimal magnetic characteristics exhibit the Ba_1.2 sample calcined at the lowest temperature of 1100 °C. An increase in the calcination temperature leads to a decrease in almost all magnetic parameters except for *B*_r_ and *M*_S_. In general, the values of the magnetic parameters of Ba samples are much lower than Sr. Based on these results, another series of Ba-based samples with a smaller average particle size of around 45–50 µm was prepared. Their magnetic properties presented at the bottom part of Table 4 were improved and partially followed the magnetic behaviour of Sr-based hexaferrites. The Ba_5.1 sample calcined at 1300 °C appears to be optimal. However, its magnetic parameters are slightly worse than those of the Sr_7.1 powder. Similar magnetic properties of BaFe_12_O_19_ hexaferrites were presented, for example, in reference^28^, where the powders of average particle size of 35 µm were prepared by ceramic method.

The interparticle magnetic interactions described by Δ*M* function in Eq. (1) can reach positive or negative values depending on the magnetization value of the virgin curve *M*_vir_ (*H*) with respect to the *M*_up_ (*H*) and *M*_down_ (*H*) magnetizations. If the value of *M*_vir_ (*H*) is greater than the mean value of *M*_up_ (*H*) and *M*_down_ (*H*), positive interactions arise related to the exchange coupling among particles. In the opposite case, Δ*M* (*H*) < 0 and negative dipolar interactions, produced by the magnetic moment of each particle, dominate. The complicated shape of the virgin curve shown in Fig. 5a indicates that both types of interactions will be present in the prepared powder samples. The obtained Henkel plots of Sr- and Ba-based hexaferrites are compared in Fig. 6. It is evident that the exchange-coupling interactions observed at small magnetic fields *H* gradually turn into dipolar interactions with increasing *H*. Such magnetization behaviour is typical also for NdFeB hard magnetic nanoparticles^36^ and nanocomposite magnets^37^. The strongest positive (Δ*M*_max_) and negative (Δ*M*_min_) interactions and the corresponding magnetic fields at which these interactions arise are listed in the right-hand columns of Table 5.

Similar shapes of the Henkel plots depending on the calcination temperature show Sr-based samples (Fig. 6a) and Ba-based samples with an average particle size of 45–50 µm (Fig. 6c). These results agree with hysteresis loop measurements. Optimal samples Sr_7.1 and Ba_5.1 calcined at 1300 °C have the strongest positive interactions shifted towards higher *H* and the negative interactions towards lower *H*. The strength of the interactions is variable and depends not only on the calcination temperature but also on the particle size.

The obtained values of magnetic properties are very similar to the study by Qiang et al.^38^. In their research, Rianna et al. synthesized SrFe_12_O_19_ from iron sand with inferior magnetic properties^39^. Also, the XRD results were comparable to the results of these studies. Manchón-Gordón et al. got, during reactive flash sintering of SrFe_12_O_19_ ceramic permanent magnets, similar values of structural data and saturation magnetization but much higher values of maximum energy product (BH)_max_^40^.

## Conclusions

The conclusions of the applied research show that even with economically optimized production, it is possible to achieve satisfactory magnetic properties of ferrite powder for producing hard ferrite magnets. Further research can be continued, e.g. by doping additive elements into the ferrite powder. The main conclusions of the influence of calcination temperature and particle size distribution on the physical properties of hexaferrite powders study are as follows:

- The SrFe_12_O_19_ and BaFe_12_O_19_ hexaferrites were successfully prepared by mixing and granulation from Fe_2_O_3_ and SrCO_3_ (BaCO_3_). Additional calcination for 2 h in the temperature range 1100–1300 °C and final crushing and milling to an average particle size of about 80–90 µm was used.
- Observations during sample preparation and from the SEM images showed that replacing strontium carbonate with barium carbonate is more advantageous. Better flow properties of BaCO_3_ and the ease of achieving a higher degree of homogeneity with the desired concentration throughout the volume were observed.
- The morphology of prepared hexaferrites revealed agglomerates consisting of large and small hexagonal-like platelet particles and semi-circular polyhedral particles. The XRD results detected the presence of two phases: dominant SrFe_12_O_19_ / BaFe_12_O_19_ and minor Fe_2_O_3_. The amount of hematite in Ba-based samples was only around 2 wt% and in Sr-based ferrites between 7 and 12 wt%. The highest amount of hematite (about 39 wt%) was observed in the reference sample.
- The magnetic properties of prepared SrFe_12_O_19_ powders with a mean particle size of about 80–90 µm are comparable to the reference sample Sr_1. The sample calcined at 1300 °C achieved better magnetic parameters from the *B-H* curve, especially (*B-H*)_max_, than the reference sample. On the other hand, the highest value of *H*_cj_ parameter is obtained for the reference sample, and the magnitude of *H*_cj_ rather decreases with increasing calcination temperature. All laboratory-prepared samples have higher saturation magnetization than the reference sample due to a markedly lower amount of hematite.
- While maintaining the mean particle size, replacing strontium with barium led to the marked deterioration of the magnetic properties of prepared powders. A sample calcined at 1100 °C appeared to be optimal.
- A partial improvement in the magnetic properties of the Ba-based powders was achieved by reducing the mean particle size to 45–50 µm. The samples calcined above 1200 °C have magnetic parameters comparable to those of the reference sample, except for *H*_cj_ and *M*_S_.
- The Henkel plots revealed a predominance of exchange-coupling (positive) and dipolar (negative) magnetic interactions at lower and higher applied magnetic fields. The strength of both types of interactions is variable, and as the calcination temperature increases, the positive and negative interactions shift to higher and lower magnetic fields.

## Data Availability

The data used in this study is available at: https://doi.org/10.5281/zenodo.10890728 and https://doi.org/10.5281/zenodo.10890791.
